# Multiplexed CRISPR-Cas9 protocol for large transgene integration into the *Schistosoma mansoni* genome

**DOI:** 10.1016/j.xpro.2024.102886

**Published:** 2024-02-13

**Authors:** Wannaporn Ittiprasert, Max M. Moescheid, Victoria H. Mann, Paul J. Brindley

**Affiliations:** 1 Department of Microbiology, Immunology and Tropical Medicine, School of Medicine and Health Sciences, George Washington University, Washington, DC 20037, USA; 2 Institute of Parasitology, Biomedical Research Center Seltersberg (BFS), Justus Liebig University, 35392 Giessen, Germany

**Keywords:** Model Organisms, Molecular Biology, Gene Expression, CRISPR

## Abstract

Precise, on-target CRISPR-Cas9 genome editing has been shown in *Schistosoma mansoni*, involving both non-homology end joining and homology-directed repair pathways. Here, we present a multiplexed CRISPR-Cas9 protocol for large transgene integration into the *S. mansoni* genome. We describe steps for deploying multiplexed ribonucleoprotein complexes (RNPs) and donor DNA preparation. We then detail procedures for introducing RNPs into schistosome eggs by square-wave electroporation in the presence of a 5′ phosphorothioate-modified double-stranded donor transgene.

For complete details on the use and execution of this protocol, please refer to Ittiprasert et al. (2023).^1^

## Highlights

• Simplify DNA donor preparation for optimal yield and its stability
• Ribonucleoprotein complex of Cas9 enzyme and gRNA assembly
• Co-electroporation of multiple RNPs and dsDNA donor into the cells
• Instructions on genotyping for transgene integration following transfection of eggs

## Abstract (editor highlights)

Publisher’s note: Undertaking any experimental protocol requires adherence to local institutional guidelines for laboratory safety and ethics.

## Graphical abstract

**Figure:**
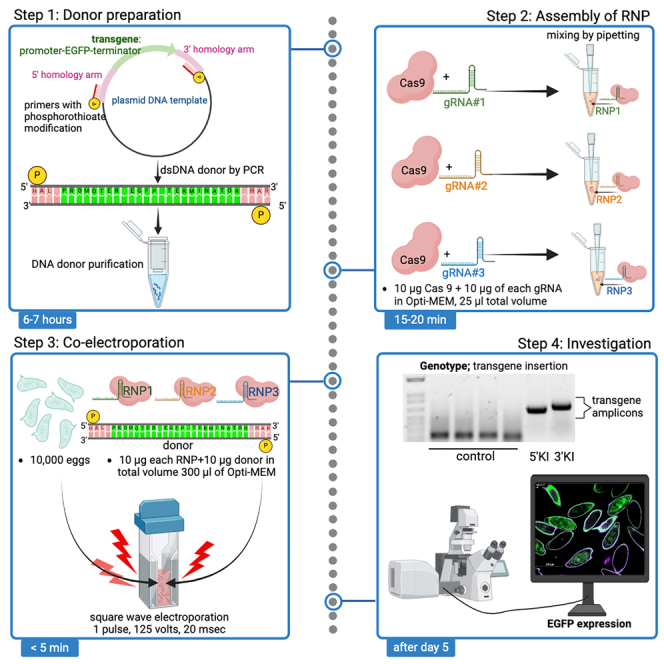

## Before you begin

### Schistosome eggs

**Timing: 1 day**

Where electroporation is employed to introduce the CRISPR/Cas-material into eggs of *S*. *mansoni,* it is necessary to isolate healthy and well developing schistosome eggs. Eggs recover from the liver lobes of *S. mansoni-*infected mice^2^ no later than 1 day. The *S. mansoni-*infected mice provide by the Schistosomiasis Resource Center (SRC), Biomedical Research Institute (BRI), without cost beyond shipping charges to registered researchers with BEI Resources, https://www.beiresources.org/Catalog.aspx?f_instockflag=In+Stock%23∼%23Temporarily+Out+of+Stock&q=schistosoma (see detail in key resources table).

1. Ensure that the eggs are free of contaminating host tissues, cells and other debris, and maintain the eggs in DMEM medium containing 20% heat-inactivated fetal bovine serum at 37°C, 5% CO_2_.^1^^,^^3^
2. For transfection of schistosome eggs with CRISPR materials delivered using square wave electroporation, suspend the eggs in Opti-MEM as the electroporation medium at a concentration of 50,000 eggs/mL.

***Note:*** The mice from SRC are housed at the Animal Research Facility of George Washington University, which is accredited by the American Association for Accreditation of Laboratory Animal Care (AAALAC no. 000347).

### Plasmid construct coding for the donor transgene/repair template

**Timing: 2 days**

To prepare the donor repair template, a custom designed plasmid, which can be synthesized, constructed and nucleotide sequence must be proved by Sanger direct sequencing. The plasmid’s cargo includes the nucleotides of the homology arms, an appropriate gene promoter, a transgene, e.g., the EGFP reporter, and a cognate terminator sequence, and serves as the PCR template for dsDNA donor synthesis using a high fidelity Taq DNA polymerase.

3. Generate dsDNA donor template using primers including five nucleotides at the 5′ end with phosphorothioate (P) modification.
4. Purify PCR amplicon by column purification kit, for example, NucleoSpin Gel and PCR Clean up (Takara) and Monarch PCR and DNA Clean up Kit (NEB).
5. Assess the concentrated and purified dsDNA donor by spectrophotometry (NanoDrop, Thermo Fisher). DNA donor length and integrity confirm by visualizing following using ethidium-stained agarose gel (1%) electrophoresis.

***Note:*** P bond substitutes a sulfur atom for a non-bridging oxygen in the phosphate backbone of the oligo. This modification renders the inter-nucleotide linkage resistant to nuclease degradation inside the cell. Hence, primers are introduced with P bonds at 5 nucleotides at their 5′-ends, which impede exonuclease degradation.

### Stock solutions

**Timing: 30 min**

6. Prepare reagents for the formation of ribonucleoprotein complexes (RNP). Perform these steps on wet ice.
  a. Dilute the Cas9 nuclease (Alt-R *Streptococcus pyogenes* Cas9 protein, Integrated DNA Technologies [IDT]) in phenol red-free Opti-MEM medium (Thermo Fisher Scientific), mix by pipetting to 1 μg/μL and aliquot in 0.5 mL tubes. Store at ‒80°C; avoid freeze/thaw cycles.
  b. Resuspend synthetic guide RNA (sgRNA) solution in nuclease-free duplex buffer (IDT), mix by pipetting to 10 μg/μL, and store at ‒20°C. sgRNA is stable for at least 6 months; avoid freeze/thaw cycles.
7. Dilute the dsDNA donor in Opti-MEM at a concentration of 1 μg/μL; store aliquots at ‒20°C.

### Designing the guide RNA

**Timing: 30 min**

The genome of *S. mansoni* is characterized by a high AT content (65%); therefore, it is critical to judiciously select the target binding sequence of the guide RNA (gRNA). To design the overlapping gRNAs for Cas9, the protospacer adjacent motif (PAM) site NGG can be used to introduce multiple, double strand breaks (DSB) due to the substrate sequence specificity of the Cas9 nuclease.

***Note:*** In our experience, gRNAs low in GC-content, and specific for an AT-rich locus around the expected cleavage-site result in poor or only modest at best CRISPR/Cas-efficiencies in editing of schistosome eggs. Accordingly, we presume that it may be more efficient to deploy multiple and overlapping gRNAs at an AT rich target site to generate a staggered ended DSB.

8. To enhance efficiency of the targeted gene knock-out, choose two to three overlapping gRNAs specific for the target site.
  ***Note:*** Deploying the overlapping gRNAs in unison leads to the deletion of most of genes of interest.
  a. To induce homology-directed repair (HDR) mediated integration of a transgene, choose gRNA sequences with PAM sites situated at 6–8 nt distance to other PAM to catalyze the deletion of large DNA fragments and to liberate staggered ended DSBs.
    ***Note:*** Bioinformatic tools including CHOPCHOP (https://chopchop.cbu.nib.no)^4^^,^^5^^,^^6^ and Breaking-Cas (https://bioinfogp.cnb.csic.es/tools/breakingcas/)^7^ provide assistance in the prediction of gRNAs with optimal on-target efficiency but with lack predicted off-target cleavage activity on the *S. mansoni* genome.
    **CRITICAL:** Using multiple gRNAs to generate large deletions of target DNA fragment, chose each gRNA with a similar CRISPR efficiency with the goal of preventing bias in DNA cleavage mediated by a more efficient gRNA. In addition, avoid gRNAs with predicted off-target site(s) proximal to the programmed target sites.

### DNA donor template

**Timing: 5–6 h**

Non-homologous end joining (NHEJ), rather than HDR, is generally the dominant mechanism responsible for repairing DSBs in the genome of cells when a single gRNA combined without (or even with) single-stranded oligodeoxynucleotides (ssODNs) is provided as DNA donor template.^8^^,^^9^ However, enhancing the efficiency of DSB repair carried out by HDR to knock in (KI) a large transgene can be improved by engineering a staggered DSB while, in tandem, providing a suitably prepared donor DNA template.^10^ Here, we use an example from our recent report^1^ to illustrate the KI procedure using a linear dsDNA carrying a large reporter transgene flanked by the *S. mansoni* ubiquitin gene promoter and its terminator. Our example uses the Ubi-EGFP-Ubi (total length, ∼ 4.5 kb) donor DNA template for HDR into schistosome gene safe harbor 1 (GSH1), as detailed below.

**Figure 1:**
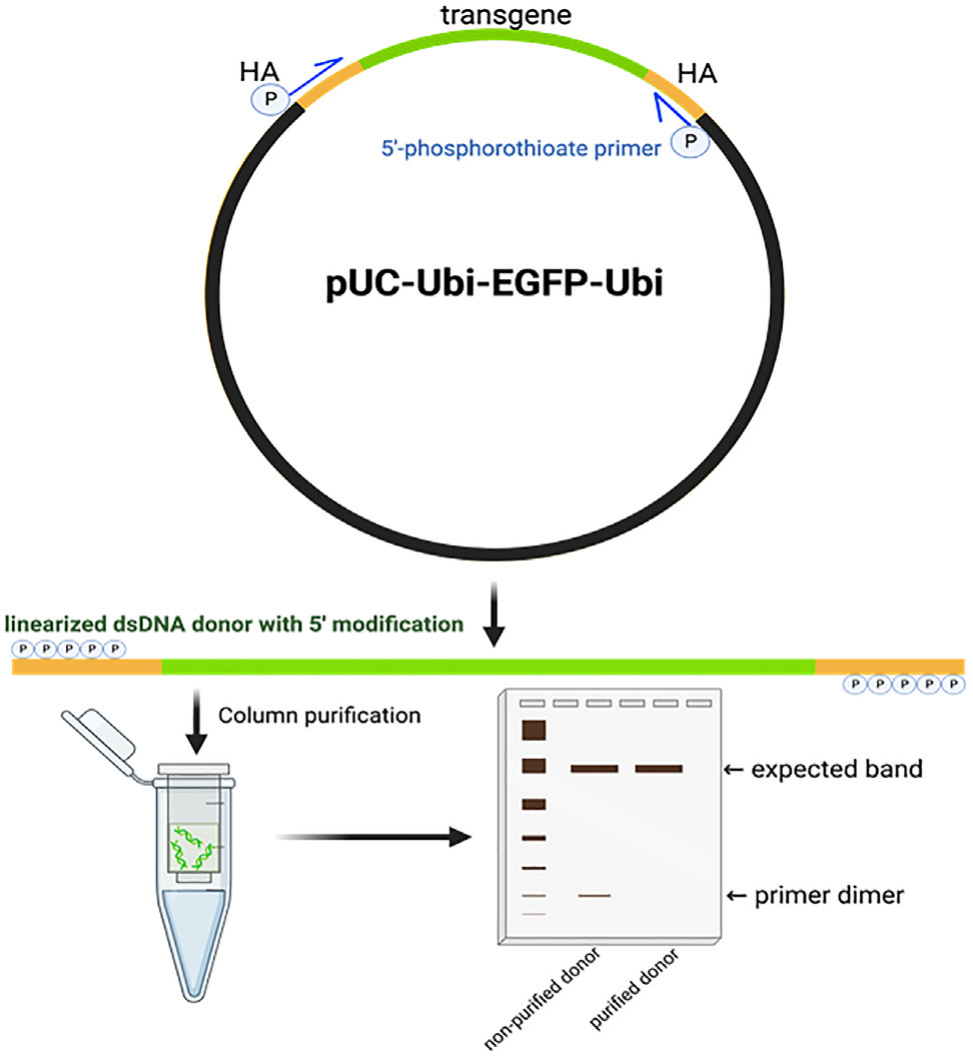
Schematic diagram of the long double stranded DNA donor Donor contains 5′phosphorothioate modification at the first 5 nt of both DNA strands. Plasmid DNA encoding gene cassette of an ubiquitin promoter driven EGFP with its terminator (green line) flanked on both sides by homology arms (HA) (yellow lines). A pair of 5′-phosphorothioated (P) primers (blue arrows) is used to amplify the dsDNA. PCR products are purified using a DNA column purification kit and inspected by agarose gel electrophoresis comparing with non-purified PCR (primer dimer contamination) amplicon. Purified DNA from the PCR, not from agarose gel, serves as the donor template for HDR.
  f. Purified dsDNA donor may be stored at ‒20°C, thawed on wet ice and warmed to 37°C before transfection (optional).
    ***Note:*** Dimers may not be visible on the agarose gel. It is helpful to use a primer analysis tool such as the OligoAnalyzer Tool from IDT and OligoEvaluator by Sigma-Aldrich to predict self-dimer or hetero-dimer formation to avoid using primers that exhibit these characteristics. If primer-dimer formation remains a concern, the PCR amplicon can be cleaned by paramagnetic beads (AMPure XP) to size-exclude shorter products (e.g., < 200 bp). This is critical purification reagent for size selected-amplicon purification. The column kit is not recommended.

9. dsDNA donor design and synthesis:
  a. Generate dsDNA donor templates using primers including 5 nucleotides at the 5′end with 5′-phosphorothioate (P) modifications.
    ***Note:*** The DNA sequence complexity and/or hairpin structure of the long dsDNA donor is critically associated with HDR efficiency. Using 5′-end-modifications with P bonds strongly enhances HDR and favors the efficiency of single-copy donor integration through retention of monomeric donor conformation. In turn, this facilitates targeted integration of the repair template (transgene) to heal the programmed Cas9-catalyzed chromosomal break.^11^
  b. Synthesized-5′-phosphorothioate-modified (≥5 nt) primers by standard desalting of 20–24 nt complementary to the desired insert, e.g., homology arms flanking promoter-enhanced green fluorescent protein gene-terminator. The 100 μM primer stock solution may be aliquoted and stored at ‒20°C. A 10 μM working solution is used to execute PCR.
  c. Perform PCRs to amplify the desired transgene donor from plasmid DNA as a template using a high-fidelity *Taq* DNA polymerase. The PCR master mix must be freshly prepared.
  d. Agarose gel electrophoresis is used to size obtained amplicons and to confirm amplification of a single fragment. If the amplicon profile seen in the gel differs from the expected product, the annealing temperature of the PCR step must be optimized to resolve this issue.
  e. Purification of the DNA donor from the PCR amplicon is accomplished using a spin-column kit, for example, NucleoSpin Gel and PCR Clean-up column (Takara) or Monarch PCR and DNA Clean up Kit (NEB). This column kit removes excess primer and/or primer dimers. The long dsDNA donor can be eluted in deionized, nuclease-free water or Opti-MEM at 70°C (Figure 1).

## Key resources table

**Table:** 

REAGENT or RESOURCE	SOURCE	IDENTIFIER
**Chemicals, peptides, and recombinant proteins**		

*Streptococcus pyogenes* Alt-R HiFi Cas9 nuclease	IDT	Cat no. 1081058
Opti-MEM	Thermo Fisher Scientific	Cat no. 31985062
DMEM	Thermo Fisher Scientific	Cat no. 11320033
Fetal bovine serum	Gibco	Cat no. A5256801
Antibiotic antimycotic	Gibco	Cat no. 15240062
Phusion high-fidelity DNA polymerase	New England Biolabs	Cat no. M0530
NucBlue Live ReadyProbes reagent	Thermo Fisher Scientific	R37605
Deoxynucleotide set, 100 mM	MilliporeSigma	Cat no. DNTP100A

**Critical commercial assays**		

AmPure XP bead-based reagent	Beckman Coulter	A63881

**Experimental models: Organisms/strains**		

Mouse liver tissues from *Schistosoma mansoni-*infected mice	Biomedical Research Institute, Rockville, MD	https://www.afbr-bri.org/schistosomiasis/

**Oligonucleotides**		

Custom guide RNA	IDT	N/A
HA-donor forward primer	This study	5′-PS bond-AGATTGCTAGAAATTTATGAAAG-3′
HA-donor reverse primer	This study	5′-PS bond-ACTGCCGAATTTATAATATTTGG-3′
Control-F	This study	TGTTATCGTCCGTCGCTTCA
Control-R	This study	GCGTTCAAACATTGCCCACT
5′ KI-F	This study	AGGGTTTTGGTTCGCAGGAT
5′ KI-R	This study	CGGAGACAATCTGGAAAGGTCA
3′ KI-F	This study	GTCCGCGTAATCGTCGTTACTA
3′ KI-R	This study	GTGGTTCCATACTATGCAGTTTCC

**Recombinant DNA**		

pUC-Ubi-EGFP-Ubi	This study	N/A

**Software and algorithms**		

CHOPCHOP		http://chopchop.cbu.uib.no/
Breaking-Cas		https://bioinfogp.cnb.csic.es/tools/breakingcas/

**Other**		

NanoDrop One	Thermo Fisher Scientific	Cat no. ND-ONE-W
DNAzol ES	Molecular Research Center, Inc.	Cat no. DN128
Electroporation cuvette	Cole-Parmer, BTX	Cat no. 45-0126
Electro Square Porator	Cole-Parmer, BTX	Model ECM830

## Materials and equipment

**Table:** PCR reaction master mix for long dsDNA donor preparation

Reagent	Amount
5× Phusion High Fidelity buffer	10 μL
10 mM dNTPs	1 μL
10 μM HA-donor forward primer	2.5 μL
10 μM HA-donor reverse primer	2.5 μL
pUC-Ubi-EGFP-Ubi plasmid DNA	variable
DMSO	1.5 μL
Phusion DNA polymerase	0.5 μL
Nuclease-free deionized water	to 50 μL
**Total**	**50 μL**

[Store Stock solution at 20°C, expiration date suggested by manufacture, the PCR reaction master mix must be freshly prepared]

**Table:** PCR cycling conditions

Steps	Temperature	Time	Cycles
Initial denaturation	98°C	30 s	1
Amplification	98°C	10 s	30 cycles
	55°C	30 s	
	72°C	3 min	
Final extension	72°C	10 min	1
	12°C	∞

**Table:** PCR master mix for transgene investigation^1^

Reagent	Amount
2× GoTaq G2 DNA polymerase mix	10 μL
10 μM forward primer (5′-KI-F or 3′-KI-R or control-F)	0.5 μL
10 μM reverse primer (5′-KI-R or 3′-KI-R or control-R)	0.5 μL
Genomic DNA	variable
Nuclease-free deionized water	to 20 μL
**Total**	**20 μL**

[Store Stock solution at 20°C, expiration date suggested by manufacture, the PCR reaction master mix must be freshly prepared]

**Table:** PCR cycling conditions

Steps	Temperature	Time	Cycles
Initial denaturation	95°C	2 min	1
Amplification	94°C	15 s	40 cycles
	58°C	30 s	
	72°C	1 min	
Final extension	72°C	10 min	1
	12°C	∞	

To optimize the PCR thermal cycling conditions, we recommended performing initial annealing with a temperature gradient, 52°C–59°C.

## Step-by-step methodological details of Cas9 mediated genome editing

### Genome editing by Cas9 mediated by multiple gRNAs

**Timing: <30 min**

**Figure 2:**
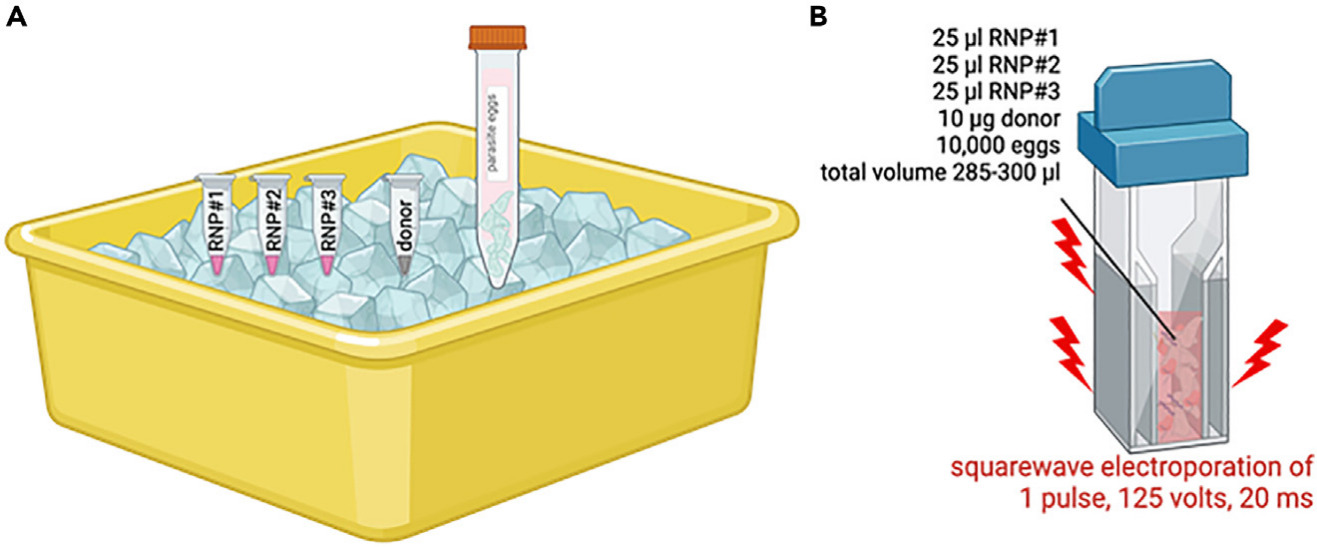
Multiple RNP preparation and co-electroporation (A) Individual, freshly prepared RNP complexes, donor, and parasite eggs in Opti-MEM, which are held on wet ice until electroporation. (B) Electroporation proceeds at 23°C–28°C. It is not necessary to pre-chill the cuvette on ice.

Ribonucleoprotein complexes (RNPs) of each synthetic gRNA (sgRNA) and Cas9 nuclease must be prepared individually. The assembly of the RNP complex is performed at ambient temperature. Thereafter, retain the RNPs on wet ice until transfection by electroporation (Figure 2).

1. Prepare dsDNA donor at a concentration of 1 μg/μL in Opti-MEM.
2. Prepare each sgRNA at 1 μg/μL from a stock solution at 10 μg/μL in Opti-MEM. In this protocol the *S. mansoni* GSH1 was targeted by three sgRNAs. Therefore, gRNAs in the example shown here are designated sgRNA#1, sgRNA#2 and sgRNA#3.
3. Prepare the Cas9 working solution by diluting to a concentration of 1 μg/μL in Opti-MEM.
4. Transfer and mix 5 μL of Opti-MEM, 10 μL of sgRNA#1 (or sgRNA#2 or sgRNA#3) and 10 μL of Cas9 into a new tube by gently pipetting (do not vortex).
  a. Incubate at ambient temperature for 15 min.
  b. Subsequently, keep RNP on wet ice until used for electroporation.
5. Using a pipette, dispense 200 μL of schistosome eggs (50,000 eggs/mL of Opti-MEM, as described above) into a 4 mm gap electroporation cuvette and add 10 μL of 1 μg/μL of the dsDNA donor, 25 μL of each RNP. Total volume in the cuvette will be ∼285 μL–300 μL.
6. Immediately proceed with square wave electroporation of a single pulse of 125 V for 20 ms (msec).
7. After electroporation, maintain the schistosome eggs in DMEM supplemented with 20% FBS, 2% antibiotic-antimycotic (penicillin-streptomycin-amphotericin B) at 37°C, 5% CO_2_. Change the culture medium daily (this is necessary) until the experiment terminates.

***Note:*** We recommend preparing a fresh working concentration of gRNA in Opti-MEM. Promptly use the RNPs for electroporation after the preparation and assembly of the RNP complex. The electroporation cuvette can be used at 23°C–28°C. A 1:1 ratio sgRNA:Cas9 at 10 μg of each reagent may be optimal, based on our studies using a range of 5 μg–20 μg of gRNA and Cas9. Although we have not seen a positive correlation between enhanced efficiency of editing and the increasing concentrations of the RNP reagents, we have not titrated the concentration of the dsDNA donor with respect to efficiency of HDR. We have observed, however, that less donor DNA resulted into a comparably lower efficiency of HDR.

**CRITICAL:** Prepare each RNP complex individually. Attempting to assemble the complexes of all the gRNAs and the Cas9 nuclease in a same tube may result in inconsistent complex assembly of the RNP and its component Cas9 and individual gRNAs. Given that schistosome eggs settle rapidly to the bottom of the cuvette, ensure that the eggs are homogeneously suspended in the medium during electroporation by frequent, gentle agitation by hand.

**Figure 3:**
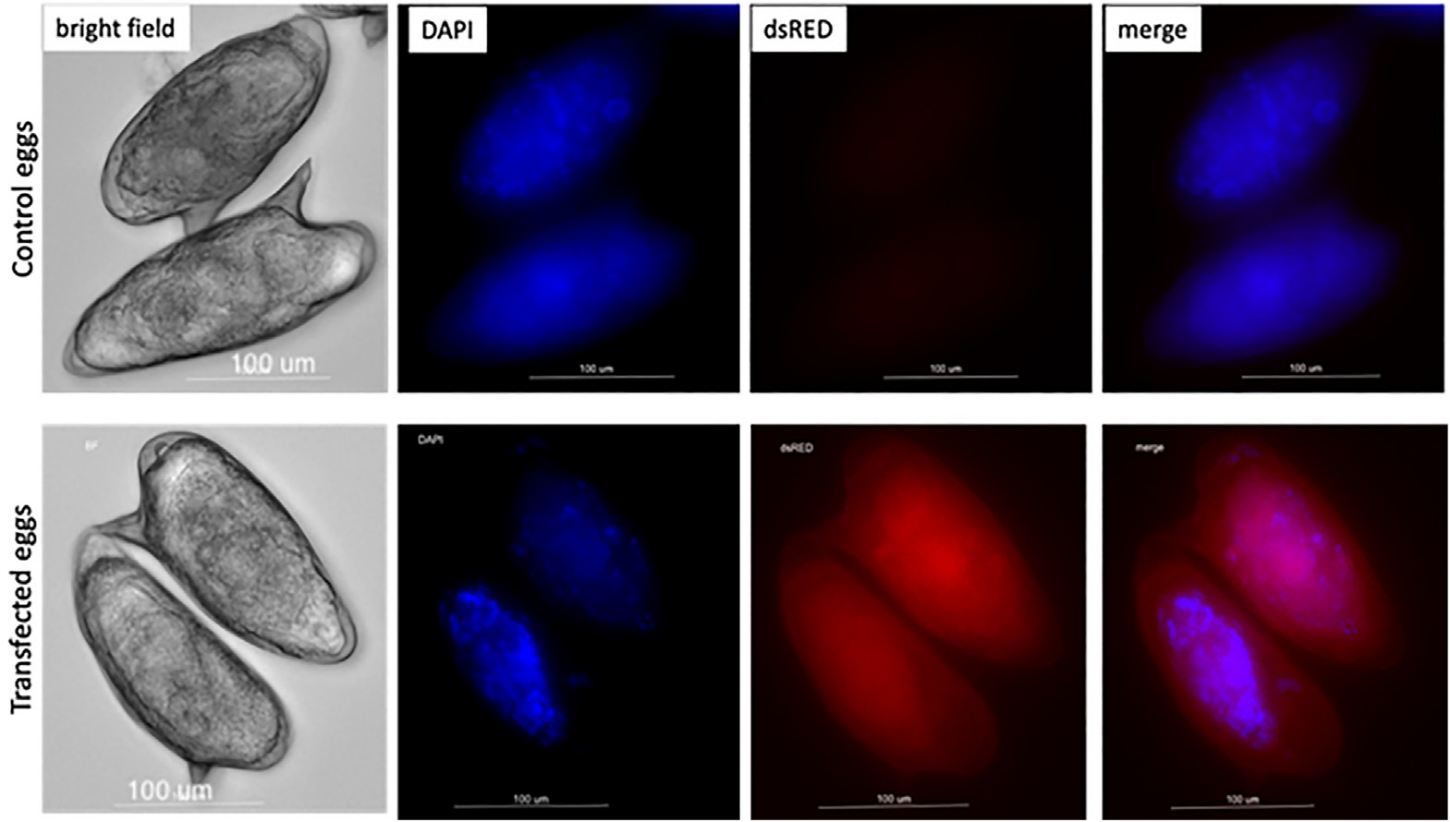
RNPs entry into parasite eggs Delivery of RNPs into schistosome eggs containing a fully developed larva tracked by RFP-tagged Cas9 enzymes (IDT). Only RNP-electroporated eggs showed positive red fluorescence (lower panels). Vital cells have been stained using by live cell staining, Hoechst 33342 (blue). The RNP delivery into larval cells was visualized using the ReadyProbes Cell viability imaging kit, Blue (Invitrogen). Control treatment group eggs did not show the red fluorescent protein (RFP) signal.

***Alternative:*** Tracking the entry of RNPs into the schistosome egg and its cells may be monitored using red fluorescent protein (RFP)-tagged Cas9 nuclease without negatively impacting CRISPR/Cas efficiency (Figure 3). Fluorescence from the tagged Cas9 is apparent up to 48 h post electroporation.

### Analysis for transgene integration

**Timing: 1–2 days**

**Figure 4:**
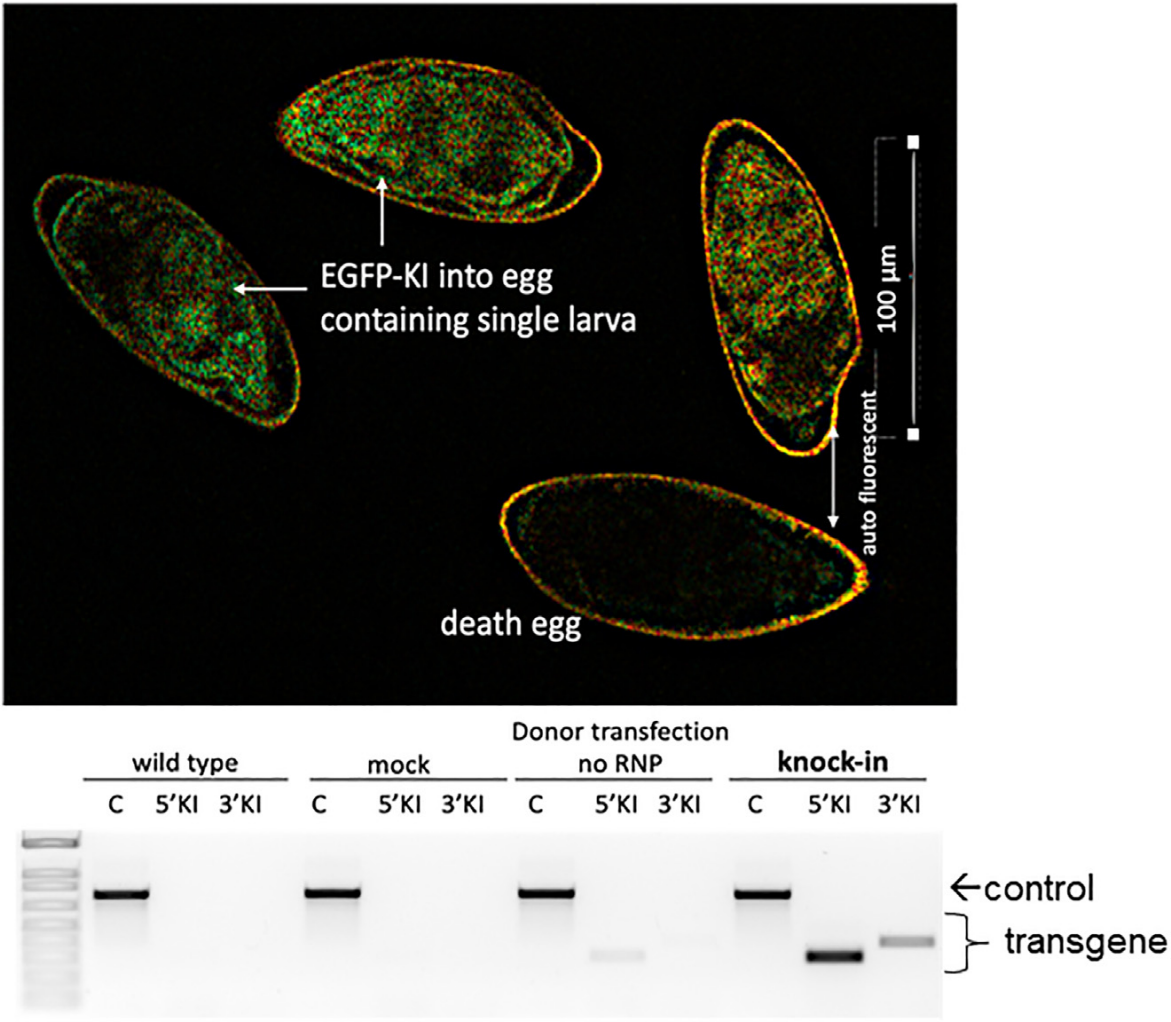
Fluorescent reporter gene knocked-in into schistosome GSH1 EGFP expression driven by the ubiquitin promoter following Cas9 mediated transgene knock-in, programmed using multiple, overlapping gRNAs. Green fluorescent protein signal seen in viable eggs (green arrows) but not in non-viable eggs (which exhibit only auto fluorescence [AF] as the white arrow indicates) beyond day 5 post electroporation (top panel as published in Ittiprasert et al., 2023^1^ as Figure 4B). Genotyping for transgene integration using PCR with primers specific for both the 5′KI and 3′KI integrations at the programmed target site. The PCR control shows positive bands in all DNA samples (bottom panel).

The genotyping for transgene integration can be performed at day 7 following transfection of the eggs. Our experience revealed that the P-modified dsDNA donor is stable in cells of the cultured schistosome eggs for as long as 5–7 days if not incorporated by HDR. We have reported EGFP expression (Figure 4, left panel) in the developing embryo within the eggshell. The transgene fluorescence displayed in these eggs could only be differentiated at day 5 post and onwards after electroporation from signals from control group eggs transfected only with the donor repair template (i.e., without RNPs). The EGFP signal from these control group eggs was no longer detected by day 10.^1^ Hence, we recommend proceeding to genome sequence/genotyping analysis at or later than 10 days after transfection to avoid false positive and cross-reactive PCR from residual donor DNA repair template (Figure 4, right panel).

8. Retrieve the eggs from the culture for analysis. Wash eggs 3 times in 1× PBS, then extract genomic DNA from the eggs using DNAzol ES, following the manufacturer’s protocol.
9. To avoid false positive PCRs due to mosaicism, locate one of the primers used for genotyping outside of the homology arms (at least 50 nt away).

***Note:*** This should prevent or at least minimize amplification from transiently retained (episomal) donor DNA molecules. We used Primer-Blast for primer design (https://www.ncbi.nlm.nih.gov/tools/primer-blast/index.cgi?LINK_LOC=BlastHome) to predict precise knock-in at the *S. mansoni* GSH1 target site. *In silico*, none of the primers cross amplify with the wild type GSH1 locus and/or elsewhere in the schistosome genome using primer pairs specificity checking parameters with database settings of ‘nr’ and exclude ‘Schistosomatidae’.

10. Undertake PCR as above using 5′ KI or 3′ KI specific primer pairs and genomic DNA of the eggs as DNA template. Also perform a positive control PCR assay for the integrity of the genomic DNAs using samples of the gDNA from each treatment and control group. The PCR master mix must be freshly prepared.

## Expected outcomes

CRISPR/Cas9-mediated editing of a target locus using multiple RNPs and overlapping gRNAs in tandem was used to improve precise transgene integration into the AT-rich *S. mansoni* genome. Large gene deletions, as well as the transgene integration of a fluorescence (EGFP) reporter, could be accomplished with higher efficiency compared to a single gRNA approach. The stability of the dsDNA donor was significantly increased by 5′-phosphorothioate modification with homology arms of <1 kb in length. In this example, homology arms of 600 bp enabled HDR insertion of a 3 kb transgene into a predicted schistosome genome safe harbor termed GSH1. Moreover, HDR efficiency also was improved.^1^ The optimized conditions to deliver CRISPR materials by electroporation into the schistosome egg were sufficiently gentle such that growth and development of the embryo within the treated egg proceeds apparently in the same way as the control, wild type eggs. This method is advantageous for functional studies when using CRISPR/Cas approaches, including for transgene gain-of-function functional genomics. We anticipate that this protocol not only will benefit research by other labs studying schistosomes but will be modifiable for other species including other helminths with AT-rich genomes.

## Limitations

It can be challenging to integrate large dsDNA donors (>3 kb) containing a transgene flanked by homology arms) into the genome. To minimize this hurdle, select/design the DNA repair donor without low DNA complexity, repetitive sequence(s) and/or self-hairpin forming loop(s). For the design of the donor sequence, non-frameshift-based processing can be considered to eliminate the above problems.

## Troubleshooting

### Problem 1

As soon as possible following electroporation, culture the eggs in 20% FBS, DMEM.

Related to this, the presence of non-viable parasite eggs, which can occur following from the egg preparation, confounds the investigation because larval development after electroporation does not proceed normally.

### Potential solution

• Use freshly prepared eggs (< 2 days in culture).
• Change egg culture medium regularly.

### Problem 2

No transgene insertion.

### Potential solution

• Ensure the integrity of the donor by visualizing following agarose gel electrophoresis. If degradation is observed (a smear rather than a single band of the correct size), prepare a fresh aliquot of the donor template.
• Contamination by primer dimers or partial donor degradation inhibits HDR efficiency.
• Optimize the assembly of the RNP complexes and the synthesis of the donor DNA template as suggested in this method. Excess quantities of non-parasite material including the Cas9 nuclease and donor repair template DNA can be toxic. By contrast, insufficient concentration of CRISPR/Cas reagents may result in sub-optimal outcome, including inefficient programmed target site cleavage and subsequent HDR.

### Problem 3

Background EGFP signals from residual/non-integrated dsDNA donor in transcript reporter transgene expression study.

### Potential solution

• Ensure to set up an experimental group, where eggs are only transfected with the dsDNA donor (no RNPs). Information from this group is used for normalization.
• For the 5′-phosphothioate modified-donor, stability within the parasite cells up to 5 days has been shown. To eliminate/minimize the bias of EGFP expression from non-integrated donor, the downstream experiments for both genotyping and EGFP expression should be undertaken only at day ≥5 after transfection.

## Resource availability

### Lead contact

Further information and requests for resources and reagents should be directed to and will be fulfilled by the lead contact, Paul J. Brindley (pbrindley@gwu.edu).

### Technical contact

Technical questions about this protocol should be directed to the technical contact, Wannaporn Ittiprasert (wannaporni@gwu.edu).

### Materials availability

All materials are available upon reasonable request.

### Data and code availability

This report did not generate or analyze any data sets.
